# Intraoperative hypotension, oliguria and operation time are associated with pulmonary embolism after radical resection of head and neck cancers: a case control study

**DOI:** 10.1186/s12871-021-01521-4

**Published:** 2021-12-03

**Authors:** Xuan Liang, Xiaohong Chen, Guyan Wang, Yue Wang, Dongjing Shi, Meiyi Zhao, Huachuan Zheng, Xu Cui

**Affiliations:** 1 Department of Anesthesiology, Beijing Tongren Hospital, Capital Medical University, Beijing, 100730 China; 2 Department of Otolaryngology Head & Neck surgery, Beijing Tongren Hospital, Capital Medical University, Beijing, 100730 China; 3 Department of Experimental Oncology, Shengjing Hospital of China Medical University, Shenyang, 110004 China

**Keywords:** Anesthesia, general, Fluid therapy, Malignant head and neck tumors, Hypotension, Pulmonary embolism

## Abstract

**Background:**

Postoperative pulmonary embolism (PE) is a serious thrombotic complication in the patients with otolaryngologic cancers. We investigated the risk factors associated with postoperative PE after radical resection of head and neck cancers.

**Methods:**

A total of 3512 patients underwent head and neck cancers radical resection from 2013 to 2019. A one-to-three control group without postoperative PE was selected matched by age, gender, and type of cancer. Univariate analyses were performed for the perioperative patient data including hemodynamic management factors. Conditional logistic regression was used to analyze the factors and their odds ratios.

**Results:**

Postoperative PE was prevalent in 0.85% (95%CI = 0.56–1.14). Univariate analyses showed that a high ASA grade, high BMI, and smoking history may be related to postoperative PE. There was significantly difference in operation time between the two groups, especially for> 4 h [22(78.6%) vs 43(51.2%), *P* = .011]. The urine output was lower [1.37(0.73–2.21) ml·kg^− 1^·h^− 1^ vs 2.14(1.32–3.46) ml·kg^− 1^·h^− 1^, *P* = .006] and the incidence of oliguria was significantly increased (14.3% vs 1.2%, *P* = .004) in the PE group. Multivariable conditional logistic regression showed postoperative PE were associated with the cumulative duration for intraoperative hypotension (OR = 2.330, 95%CI = 1.428–3.801, *P* = .001), oliguria (OR = 14.844, 95%CI = 1.089–202.249, *P* = .043), and operation time > 4 h (OR = 4.801, 95%CI = 1.054–21.866, *P* = .043).

**Conclusions:**

The intraoperative hypotension, oliguria, and operation time > 4 h are risk factors associated with postoperative PE after radical resection of head and neck cancers. Improving intraoperative hemodynamics management to ensure adequate blood pressure and urine output may reduce the occurrence of such complications.

## Background

Head and neck cancers are common cancers in otorhinolaryngology head and neck surgery. In China, head and neck cancer ranks ninth in the incidence of malignant tumors, sixth in males, and is the seventh leading cause of death among all tumors [1]. In the United States, there will be 53,260 estimated new cases of head and neck cancers, and 10,750 patients will die of such diseases in 2020 [2]. Radical resection is the first choice for patients affected with these types of cancers. In the past few years, the thrombotic complications in patients with head and neck cancers after radical resection have received considerable attention, especially pulmonary embolism (PE).

PE is the sudden blockage of the pulmonary artery or its branches by an embolus from the venous system or the right heart, which is characterized by dysfunction of the pulmonary circulation and respiratory system. Reported studies suggest that the incidence of this complication varies between 0.05 and 2.17% in otolaryngologic diseases [3–6]. In a recent survey in our hospital, the incidence and mortality of postoperative PE in head and neck cancers patients were 0.37 and 0.11%, respectively [7]. Although the incidence of this complication is low, the consequences are serious as they may lead to the extension of hospitalization time, the increase of hospitalization expenses, the disability or even death of patients. Additionally, due to its low incidence it is easy to be ignored by anesthesiologists and surgeons. Since many previous studies have not distinguished the risk of PE in patients, the reported incidence estimates may be underestimated. A recent study showed that the incidence of deep venous thrombosis (DVT) or PE in otolaryngology patients with high risk of thrombotic complications was similar to that in general surgery, up to 1.5–13% [8, 9]. A review of the literature, revealed that very few studies have evaluated the risk factors of DVT and PE in head and neck cancer surgery. Factors such as advanced age, obesity, high Caprini scale, and red cell transfusion may pose a profound impact in otolaryngology patients [10–12]. In addition, head and neck surgery has many special features may increase PE risk, such as long operation time, the veins in the neck may be injured by neck dissection, bandaging the neck or tracheotomy may increase immobilization time. The only possible effective intervention is the preventive application of thromboprophylaxis, but due to concern for hemorrhagic complications, their perioperative applications are limited. There are still few reports on the perioperative risk factors of postoperative PE after radical resection of head and neck cancers, especially those related to perioperative anesthesia management. Therefore, if we can identify these risk factors, it would be worth to determine whether we can actively adjust the perioperative anesthesia management strategies to reduce the incidence of postoperative PE in such patients.

In the current study, we tested the hypothesis that postoperative PE is associated with intraoperative hypotension, urine output or operation time. We conducted a case-control study, examining all patients underwent radical resection with head and neck cancers who suffered postoperative PE during a 6 yr period at Beijing Tongren Hospital.

## Methods

### Design and subjects

This study was approved by the Institutional Review Board (IRB) at Beijing Tongren Hospital. Because patients were not subjected to investigational actions and no identified data would be used, the requirement for written informed consent was waived. The full name of IRB which waived the need for written informed consent is “The Ethics Committee of Beijing Tongren Hospital, Capital Medical University”.

We conducted a retrospective case-control study, all patients who underwent head and neck cancers radical resection at Beijing Tongren hospital from January 2013 to October 2019 were screened in the hospital’s database system for a diagnosis of “pulmonary embolism” independently by an anesthesiologist and an otolaryngologist. Since the searches were independently conducted by two researchers, we are confident that all the patients were included. Subsequently, we excluded patients for whom complete medical records could not be accessed or who had DVT or PE prior to the surgery.

### Variables recorded

Perioperative patient data were collected from medical records. All the electronic medical records were reviewed by an anesthesiologist and an otolaryngologist to ensure the authenticity and accuracy of the data. These data consisted of patients’ age, gender, ASA grade, past medical history, preoperative laboratory data, location and histology of the cancers, date of operation, operative details such as occurrence and duration of intraoperative hypotension, intraoperative fluids given, urine output, operation time, and whether the intensive care unit (ICU) was required. Outcomes of patients were assessed according to the length of hospital stay and expense of hospital at discharge.

Intraoperative hypotension was defined as systolic blood pressure (SBP) < 90 mmHg, or mean blood pressure (MBP) < 65 mmHg, or relative to the baseline(> 20% decrease from the baseline) [13]. Considering the transient high blood pressure caused by the tension after entering the operating room, the baseline blood pressure was defined as the blood pressure measured at preoperative evaluation the day before surgery. Oliguria was defined as intraoperative urine output< 0.5 ml·kg^− 1^·h^− 1^.

### Control match

**Fig. 1:**
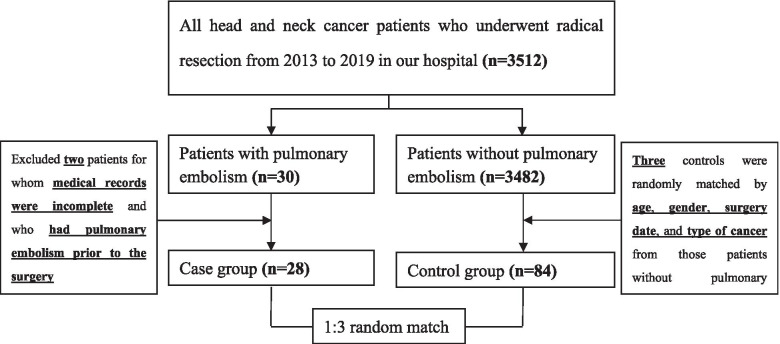
Flow chart for match procedure for patients’ selection

Age (within 5 yr), gender, surgery date (within 1 yr), and type of cancer were selected as our baseline factors to match control subjects. For each case, we used random number table to randomly match three controls who met the matching criteria in all 3482 patients without pulmonary embolism. If there are less than three eligible matches, we will appropriately relax the match restrictions. The match procedure for patients’ selection is shown in Fig. 1**.**

### Statistical analysis

Categorical variables are reported as frequency and percent. The rates of occurrence of perioperative hypotension were compared using the χ^2^ test and are presented as odds ratios (ORs) for postoperative PE and 95% confidence intervals (CIs). Normally distributed continuous variables are reported as the mean (SD) and compared using the t-test. Continuous variables, not normally distributed, are reported as the median with the interquartile range representing the difference between the 25th and 75th percentiles and compared using the Mann-Whitney test. The factors with statistically significant differences in univariate analysis and their ORs were further determined by conditional logistic regression. We used a receiver operating characteristic (ROC) curve to determine the specificity and sensitivity of urine output and cumulative duration of hypotension for predicting postoperative PE and calculate the area under the curve (AUC). The best cutoff value was obtained by maximization of the Youden index. A significance level of *P* ≤ 0.05 was used for each hypothesis. The statistical analysis was performed with the SPSS software version 25(IBM Corp., Armonk, NY, USA).

## Result

Among 3512 patients with head and neck cancers who underwent radical resection, the baseline characteristics of all patients were presented in Table 1. There were 30[0.85%(95%CI = 0.56–1.14)] cases of postoperative PE according to the data in hospital’s database system. Two patients were excluded for incomplete medical records, so the total number of patients that were analyzed was 28 with postoperative PE and a matched control group of 84 patients. No intraoperative diuretics were used in all patients. Sequential compression devices were routine used in all patients after surgery. Except for one patient who died of multiple organs failure due to PE, no other systemic complications such as renal injury, myocardial injury were observed. These data were also confirmed by hospital medical records and discharge diagnosis.

**Table 1:** The baseline characteristics of all 3512 patients

Characteristic	Patients with PE (***n*** = 30)	Patients without PE (***n*** = 3482)	***P*** value
Age(y)	63.6 (10.1)	58.9 (17.4)	.003
Gender [No. (%)]			
Female	6 (20.0%)	426 (12.2%)	.201
Male	24 (80.0%)	3056 (87.8%)	.201
Weight (kg)	70.3 (9.8)	69.8 (12.0)	.824
Operation time (h)	5.49 (2.15)	2.85 (1.81)	<0.0001

**Fig. 2:**
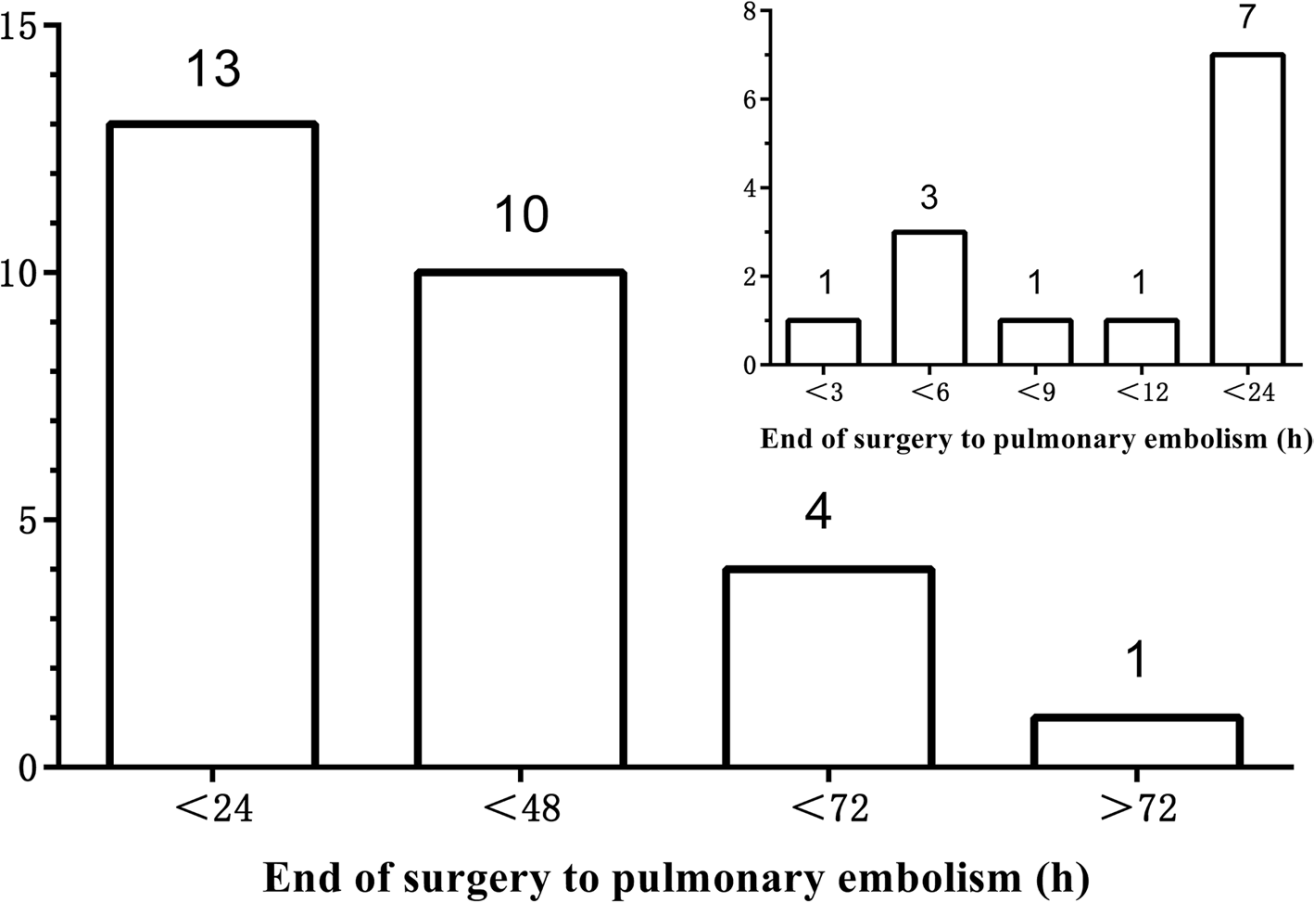
Temporal distribution of postoperative PE episodes for all the 28 PE patients. Approximately 80% of the postoperative PE < 48 h after surgery

The baseline demographic characteristics of the postoperative PE and the control groups are shown in Table 2. The PE group had a higher BMI [24.6(3.2) vs 23.2(3.2), *P* = .038], and ASA grade III was more common (25.0% vs 7.1%, *P* = .011). Laryngeal carcinoma (64.3%)was more prevalent among the patients with PE than other cancers. The baseline blood pressure in the PE group was higher than in the control group [SBP:134 (15) vs 124 (17), *P* = .005, MBP:98 (9) vs 92 (9), *P* = .005]. The PE group had a higher serum Cr levels [75.8(12.8) vs 68.9(15.3), *P* = .038], but both groups were within normal levels. More patients in the PE group had a history of smoking (67.9% vs 45.2%, *P* = .038). All patients had normal coagulation function before the operation. Whether Caprini score or Charlson comorbidity index, no significant difference was founded between the two groups. Table 3 showed the intraoperative and postoperative characteristics of tow group, the operation time of patients in the PE group was significantly longer than that of control group [5.49(2.15) vs 4.39(1.74), *P* = .007]. In addition, more patients in the PE group experienced> 4 h operation [22(78.6%) vs 43 (51.2%), *P* = .011]. There was no significant difference between the two groups in intraoperative infusion of crystal fluid or colloidal fluid [Crystalloid:5.60(4.47–6.68) ml·kg^− 1^·h^− 1^ vs 6.25(4.92–8.23) ml·kg^− 1^·h^− 1^, *P* = .069, Colloid:1.62(1.14–2.45) ml·kg^− 1^·h^− 1^ vs 1.88(0–2.68) ml·kg^− 1^·h^− 1^, *P* = .700], but the urine output was lower in the PE group [1.37(0.73–2.21) ml·kg^− 1^·h^− 1^ vs 2.14(1.32–3.46) ml·kg^− 1^·h^− 1^, *P* = .006]. The incidence of oliguria in the PE group was higher, and the difference was statistically significant (14.3% vs 1.2%, *P* = .004). There was no difference in ICU requirement (50.0% vs 41.7%, *P* = .441), but the length of the ICU stay was longer in the PE group [2.5 (1–5) days vs 0(0–1) days, *P* < .001]. Moreover, the expense of the hospital at discharge was much higher in the PE group [73,173(6699) yuan vs 37,800(2416) yuan, *P* < .001], their hospitalization was significantly longer [24 (17–26) days vs 17 (13–22) days, *P* = .002], and mortality was higher, but this difference did not reach statistical significance (3.6% vs 0.0%, *P* = .082). Figure 2 shows the temporal distribution of postoperative PE episodes for all the 28 PE patients. Approximately 80% of the PE occurred< 48 h after surgery. Vital signs were monitored in all patients after operation, and no patients developed severe or prolonged hypotension, either in the otolaryngology ward or in the ICU.

**Table 2:** Comparison of Baseline Demographic Characteristics Between Patients with Head and Neck Cancer and Control Group

Characteristic	Case Group(***N*** = 28)	Control Group(***N*** = 84)	***P*** Value
Age (y)	63.6 (10.1)	62.7 (9.3)	.659
Gender [No. (%)]			
Female	6 (21.4%)	18 (21.4%)	.500
Male	22 (78.6%)	66 (78.6%)	.500
Weight (kg)	70.3 (9.8)	66.7 (11.3)	.133
Height (m)	1.69 (0.06)	1.69 (0.08)	.783
BMI	24.6 (3.2)	23.2 (3.2)	.038
ASA Grade ≥ III [No. (%)]	7 (25.0%)	6 (7.1%)	.011
Pathologic diagnosis [No. (%)]			
Laryngeal carcinoma	18 (64.3%)	54 (64.3%)	
Thyroid carcinoma	5 (17.9%)	15 (17.9%)	
Hypopharyngeal carcinoma	2 (7.1%)	6 (7.1%)	
Others	3 (10.7%)	9 (10.7%)	
Baseline blood pressure (mmHg)			
Baseline SBP	134 (15)	124 (17)	.005
Baseline MBP	98 (9)	92 (9)	.005
Preoperative laboratory data			
Hemoglobin (g litre^− 1^)	142.5 (12.8)	139.1 (14.77)	.290
Hct (%)	42.7 (3.5)	41.4 (4.2)	.163
PT (s)	11.5 (0.9)	11.2 (0.9)	.203
APTT (s)	26.4 (3.5)	26.7 (3.6)	.668
Fbg (g litre^−1^)	3.2 (0.8)	3.2 (0.9)	.903
INR	0.97 (0.07)	0.95 (0.07)	.152
Glu (mmol litre^−1^)	5.56 (0.69)	5.61 (1.60)	.878
Bun (mmol litre^−1^)	5.3 (1.4)	5.1 (1.4)	.598
Cr (mmol litre^−1^)	75.8 (12.8)	68.9 (15.3)	.038
Past medical history [No. (%)]			
Smoking	19 (67.9%)	38 (45.2%)	.038
Radiotherapy or chemotherapy	5 (17.9%)	14 (16.7%)	.884
Anticoagulation	1 (3.6%)	7 (8.3%)	.397
Tracheotomy within one week	1 (3.6%)	5 (6.0%)	.628
Caprini scale	6.5 (1.0)	6.4 (1.2)	.638
Charlson comorbidity index	4.2 (1.2)	4.0 (1.2)	.432

*Abbreviation*: *BMI* Body mass index, *ASA* American Society of Anesthesiologists, *SBP* Systolic blood pressure, *MBP* Mean blood pressure, *Hct* Hematocrit, *PT* Prothrombin time, *APTT* Activated partial thromboplastin time, *Fbg* Fibrinogen, *INR* International normalized ratio, *Glu* Glucose, *Bun* Blood urea nitrogen, *Cr* Creatinine

**Table 3:** Intraoperative and Postoperative Characteristics of Postoperative PE in Patients with Head and Neck Cancer and Control Group

Characteristic	Case Group(***N*** = 28)	Control Group(***N*** = 84)	***P*** Value
Intraoperative data			
Operation time (h)	5.49 (2.15)	4.39 (1.74)	.007
Operation time>4 h [No. (%)]	22 (78.6%)	43 (51.2%)	.011
Crystalloid (ml·kg^−1^·h^−1^)	5.60 (4.47–6.68)	6.25 (4.92–8.23)	.069
Colloid (ml·kg^−1^·h^− 1^)	1.62 (1.14–2.45)	1.88 (0–2.68)	.700
Urine output (ml·kg^−1^ h^− 1^)	1.37 (0.73–2.21)	2.14 (1.32–3.46)	.006
Oliguria [No. (%)]	4 (14.3%)	1 (1.2%)	.004
Blood loss (ml·kg^−1^ h^− 1^)	0.33 (0.16–0.72)	0.43 (0.24–0.80)	.505
Vasodilator drugs [No. (%)]	6 (21.4%)	20 (23.8%)	0.796
Postoperative data			
ICU requirement [No. (%)]	14 (50.0%)	35 (41.7%)	.441
Length of ICU stay (days)	2.5 (1–5)	0 (0–1)	<.001
Length of hospital stay (days)	24 (17–26)	17 (13–22)	.002
Expense of hospital (yuan)	73,173 (6699)	37,800 (2416)	<.001
Mortality [No. (%)]	1 (3.6%)	0 (0.0%)	.082

*Abbreviation*: *ICU* Intensive care unit

The incidence of intraoperative hypotension in the two groups is shown in Table 4. Although there was no significant difference in the absolute blood pressures or the percentage decrease from the baseline, the cumulative duration of intraoperative hypotension showed a significant difference between two group. The cumulative duration of absolute hypotension (SBP < 90 mmHg or MBP < 65 mmHg) was longer in the PE group [SBP:0.38(0–0.86) h vs 0.06(0–0.41) h, *P* = .030, MBP:0.21(0–0.50) h vs 0.01(0–0.19) h, *P* = .014]. Moreover, the percentage decrease from the baseline (> 20%) showed a similar result [SBP:2.77(0.27–4.54) h vs 0.19(0–1.27) h, *P* < .001, MBP:2.23(0.85–4.68) h vs 0.32(0–1.14) h, *P* < .001].

**Table 4:** Intraoperative Hypotension in Postoperative the PE and Control Groups

Variable	Case Group (***N*** = 28)	Control Group (***N*** = 84)	***P*** Value
Intraoperative MBP			
<65 mmHg [No. (%)]	20 (71.4%)	43 (51.2%)	.062
Cumulative duration<65 mmHg (h)	0.21 (0–0.50)	0.01 (0–0.19)	.014
Decrease from the baseline> 20% [No. (%)]	24 (85.7%)	56 (66.7%)	.053
Cumulative duration decreases from the baseline> 20% (h)	2.23 (0.85–4.68)	0.32 (0–1.14)	<.001
Intraoperative SBP			
<90 mmHg [No. (%)]	19 (67.9%)	44 (52.4%)	.153
Cumulative duration<90 mmHg (h)	0.38 (0–0.86)	0.06 (0–0.41)	.030
Decrease from the baseline> 20% [No. (%)]	24 (85.7%)	56 (66.7%)	.053
Cumulative duration decreases from the baseline> 20% (h)	2.77 (0.27–4.54)	0.19 (0–1.27)	<.001

*Abbreviation*: *MBP* Mean blood pressure, *SBP* Systolic blood pressure

Adjusted ORs by conditional logistic regression for variables evaluated for their association with postoperative PE are presented in Table 5, the factors significantly associated with postoperative PE were the cumulative duration of the MBP decreases> 20% from the baseline (OR = 2.330, 95%CI = 1.428–3.801, *P* = .001), oliguria (OR = 14.844, 95%CI = 1.089–202.249, *P* = .043), and operation time > 4 h(OR = 4.801, 95%CI = 1.054–21.866, *P* = .043).

**Table 5:** The Results of Conditional Logistic Regression

Variable	***P*** Value	OR	95%CI
Cumulative duration time of intraoperative MBP decrease from the baseline > 20%	.001	2.330	1.428–3.801
Oliguria	.043	14.844	1.089–202.249
Operation time >4 h	.043	4.801	1.054–21.866
Intraoperative MBP <65 mmHg	.535		
Cumulative duration time of intraoperative MBP<65 mmHg	.526		
Intraoperative MBP decrease from the baseline > 20%	.329		
Intraoperative SBP<90 mmHg	.300		
Cumulative duration time of intraoperative SBP<90 mmHg	.078		
Intraoperative SBP decrease from the baseline > 20%	.869		
Cumulative duration time of intraoperative SBP decrease from the baseline > 20%	.770		
Urine output	.076		
ASA grade ≥ III	.056		
History of smoking	.185		
BMI	.148		

*Abbreviation*: *OR* Odds ratio, *CI* Confidence interval, *MBP* Mean blood pressure, *SBP* Systolic blood pressure, *BMI* Body mass index, *ASA* American Society of Anesthesiologists

**Fig. 3:**
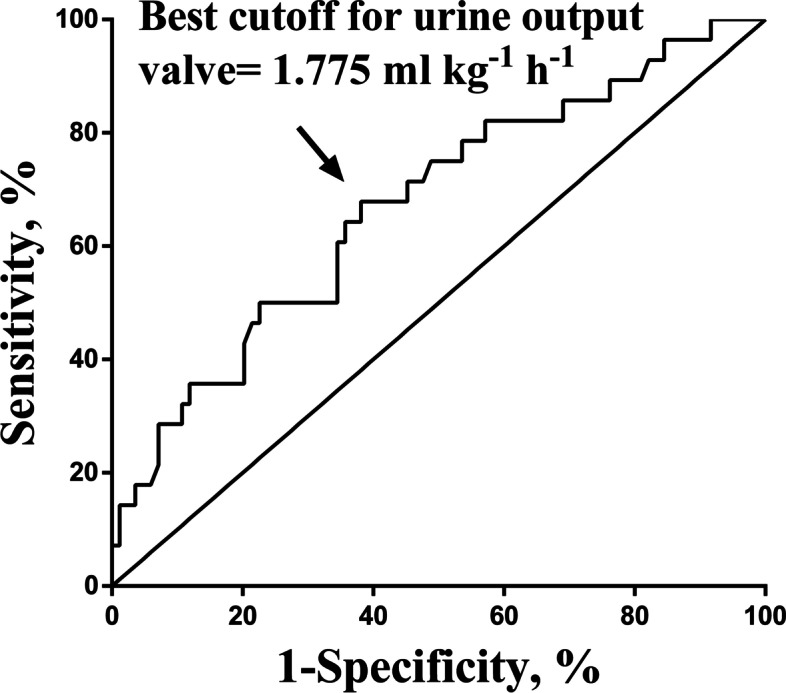
ROC curve of urine output. The AUC was derived as 0.6722, the best cutoff value of the urine output was determined as 1.775 ml kg^−1^ h^−1^, the sensitivity was 67.9% and the specificity was 61.9%

**Fig. 4:**
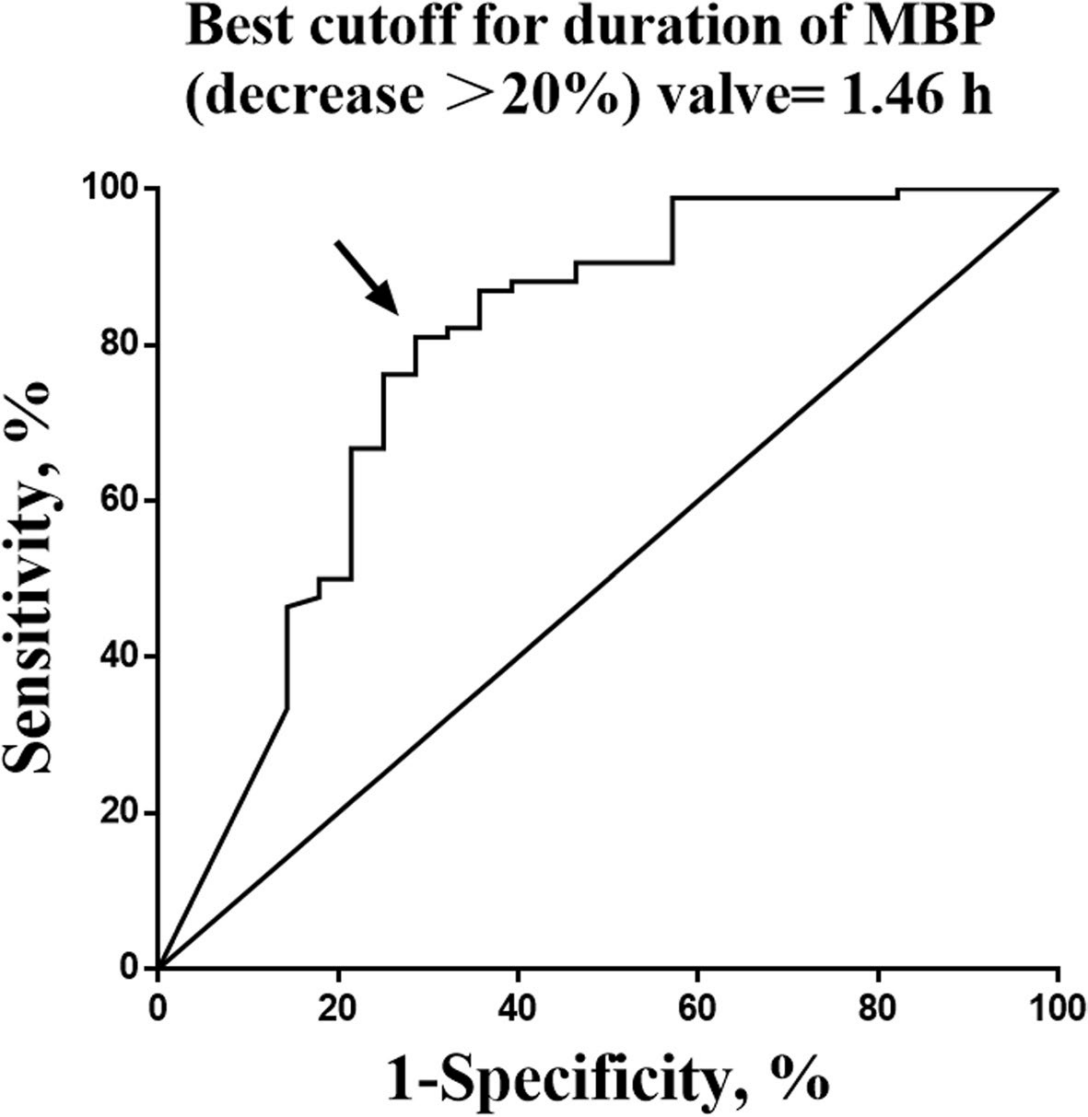
ROC curve of cumulative duration of MBP (decrease> 20%). The AUC was derived as 0.7842, the best cutoff value of the cumulative duration of MBP decrease> 20% was determined as 1.46 h, the sensitivity was 80.95% and the specificity was 71.43%

ROC curve of the urine output is shown in Fig. 3 The AUC was calculated to be 0.6722(95%CI 0.555–0.789, *P* = .007), and the best cutoff value of urine output was determined to be 1.775 ml kg^− 1^ h^− 1^ by the Youden index, the sensitivity and specificity were 67.9(95%CI, 59.3–76.5%) and 61.9%(95%CI, 52.9–70.9%), respectively. Figure 4 showed the ROC curve of the cumulative duration of hypotension (MBP decrease> 20%), the AUC was derived as 0.7842(95%CI 0.671–0.898, *P* < .000), and the best cutoff value of the cumulative duration of hypotension (MBP decrease> 20%) was determined as 1.46 h, the sensitivity was 80.95%(95%CI, 70.9–88.7%) and the specificity was 71.43%(95%CI, 51.3–86.8%).

## Discussion

Using the anesthesia database of 3512 patients at Beijing Tongren hospital, the postoperative PE was prevalent in 0.85%(95%CI = 0.56–1.14). The main finding of this study is that the intraoperative cumulative duration of hypotension, oliguria, and operation time > 4 h were significant risk factors for postoperative PE in patients underwent radical resection with head and neck cancers.

Many assessment models have been used to evaluate the risk of thrombotic disease in patients, such as the Roger score [14], Padua score [15], and Caprini scale [16], which quantitatively layer the risk in order to guide preoperative thromboprophylaxis. But the efficacy and accuracy of these models are still controversial [17]. We compared the Caprini scale and Charlson comorbidity index, the mean Caprini scale and was 6.5 in the PE group and 6.4 in the C group, all patients were at high risk of thromboembolism, but there were no significant differences was founded between the two groups. Therefore, we suspected that some other factors may also affect the occurrence of postoperative PE. In our study, we investigated some factors such as blood pressure control and fluid therapy, aiming to find some controllable factors to improve the prognosis of patients.

In general anesthesia, a 20% reduction in normal blood pressure is considered acceptable, generally. Excessive blood pressure reduction beyond that should be avoided. Intraoperative hypotension is often considered to be related to the occurrence of postoperative adverse events [18–21]. Due to the special location of the cancer, dysphagia and dietary problems often occur in patients with head and neck cancers. These patients usually have somewhat nutritional problems, preoperative dehydration, and hypovolemia are not uncommon [22]. Surgical resection of head and neck cancers results in a large trauma but is not associated with high blood loss. Using the amount of blood loss as a reference for fluid infusion, it usually leads to insufficient intake. In addition to the vasodilation effect of anesthesia, all these factors may lead to severe hypovolemia and insufficient blood perfusion in important organs during operation, which can even last until after the operation. Few studies have paid attention to the relationship between intraoperative fluid management and postoperative PE. Insufficient blood volume may lead to slow blood flow and increase blood viscosity, which may be risk factors for thrombosis. We did not find a difference in absolute or percentage blood pressure change between the PE group and the control group. However, the cumulative duration of intraoperative hypotension was significantly different between the two groups, whether absolute or relative, and the ROC curve analysis showed that the cumulative duration of hypotension (MBP decrease> 20%) had an optimal cut-off value of 1.46 h. This suggests that short-term intraoperative hypotension is not enough to lead to hemodynamic changes that may cause thrombosis, but when the cumulative time of hypotension during the perioperative period is too long it will lead to hypoperfusion, which will in turn lead to hypoxic-ischemic damage of important organs and increase the probability of complications, including thrombus complications. Mechanism of hypotension leading to thrombosis is unclear, one research has shown that orthostatic hypotension had a moderately increased risk of VTE, which may be due to changes in posture leading to vasodilation and lower extremity venous stasis, this results in a decrease in venous return to the heart or a decrease in cardiac output [23]. We suspect that intraoperative hypotension, which associates with vasodilation or low cardiac output induced by anesthesia may have a similar effect on thrombosis. Under anesthesia, physiological compensatory mechanisms such as the neurohumoral effects, the skeletal muscle pump, or neurovascular compensation may be impaired, possibly will produce a more serious consequence. Also, hypoxic-ischemic damage may lead to vascular endothelial injury and causes the blood to be hypercoagulable. Triple low state, a combination of hypotension, low bispectral index, and low minimum alveolar concentration of volatile anesthesia, was considered to increases hospital stay and perioperative mortality [24]. In a recent study, Kertai failed to found the association between cumulative duration of triple low state and perioperative complications or mortality [25]. This might be interpreted as avoiding hypotension do not have any benefit for patients, however, Kertai’s study defined hypotension as mean arterial pressure < 75 mmHg, which is not severe hypotension in clinical practice. So, the duration of hypotension is still a matter we need special care in perioperative management.

Urine output is also a commonly monitored indicator to reflect tissue perfusion during surgery. Many studies have shown the relationship between fluid infusion and postoperative complications of head and neck surgery [26–28], but few have paid attention to urine output. We found that urine output in the PE group was significantly lower than that in the control group. Corresponding, the incidence of oliguria was significantly higher in the PE group. This phenomenon may be related to the long cumulative duration of intraoperative hypotension, renal perfusion was partly affected. In multivariable conditional regression analysis, urine output was not associated with postoperative PE, but the *P* value was very close to the threshold. This may be due to the recall bias of the retrospective study, the limited sample size, and other reasons. We believe that intraoperative fluid management to ensure adequate urine output is still an important factor affecting the prognosis of thrombotic complications. Elaborate goal-directed fluid therapy will be a trend for future studies and may have potential benefits for these patients. Oliguria is a risk factor in multivariable conditional regression analysis, however, due to the large 95%CI range of its OR value, its credibility was reduced. The internationally accepted standard for oliguria is< 0.5 ml·kg^− 1^·h^− 1^, based on which there is a significant difference between the two groups. The ROC curve analysis showed that the urine output had an optimal cut-off value of 1.775 ml·kg^− 1^·h^− 1^, this may be associated with hypercoagulability and a higher requirement for fluid load in patients with malignant tumors. Due to its low sensitivity and specificity, it is not accurate to use the urine output as an independent indicator for predicting the PE after general anesthesia, postoperative PE may be the result of multiple factors.

Factors such as a high ASA grade, high BMI, smoking history, and operation time may be related to postoperative PE in this study, but these factors are uncontrollable in the short term, it is difficult to adopt active measures based on them to reduce the risk of postoperative PE. Other factors, including age, gender, tumor type, which have been identified as the risk factors for pulmonary embolism and have been included in the commonly used risk assessment system, were used as a matching condition to eliminate effects of their influence on the results.

We also assessed the outcome of patients, due to the close monitoring after the operation, most pulmonary embolism was found and treated in time, and there was no difference in mortality between the two groups. But this kind of complication increases the patient’s hospitalization time and expense, increases the use of medical resources, avoids the occurrence can economize the medical resources and reduces the medical burden.

The current study has some limitations. First, the single center, retrospective nature of the design and the relatively small number of patients with PE significantly limit the interpretation of the data. Accordingly, there is the potential for recall or selection bias, such as inaccurate blood pressure records. Second, patients with subclinical PE were not identified, and only symptomatic patients were investigated by imaging. Therefore, it is possible that our study underestimated the true PE incidence. In addition, we did not adjust for other confounding factors such as operation time, history of smoking, or surgical confounders, because the number of remaining cases after adjustment is too small to make accurate statistical analysis. We cannot rule out the possibility that the between-group differences may be due, at least in part, to the differences in the confounding factors, and only revealed a phenomenon that hypotension may be associated with PE. A postulate that hypotension and hypoperfusion are perhaps causal for PE cannot be determined with a case-control study, the relationship between the risk factors and conclusions is exploratory, further prospective or randomized controlled studies are needed to confirm this phenomenon and clarify its mechanism.

## Conclusions

our retrospective case-control study of postoperative PE obtained from 3512 patients with head and neck cancers admitted to Beijing Tongren Hospital during more than 6 yr is the largest reported study to date. We found an incidence of postoperative PE in head and neck cancers patients at our hospital is 0.85%. Cumulative duration of intraoperative hypotension, oliguria, and operation time > 4 h are associated with PE after radical resection of head and neck cancers. Appropriate perioperative anesthetic management to ensure adequate blood pressure and urine output, may be an effective measure to reduce the occurrence of postoperative PE in head and neck cancers patients, especially in the high-risk patients with long operation time.

## Data Availability

The datasets used and/or analyzed during the current study are available from the corresponding author on reasonable request.

## Abbreviations

PE: Pulmonary embolism
DVT: Deep venous thrombosis
BMI: Body mass index
ASA: American Society of Anesthesiologists
SBP: Systolic blood pressure
MBP: Mean blood pressure
Hct: Hematocrit
PT: Prothrombin time
APTT: Activated partial thromboplastin time
Fbg: Fibrinogen
INR: International normalized ratio
Glu: Glucose
Bun: Blood urea nitrogen
Cr: Creatinine
ICU: Intensive care unit
ORs: Odds ratios
CIs: Confidence intervals
ROC: Receiver operating characteristic
AUC: Area under the curve
